# Construction and validation of a pyroptosis-related gene signature associated with the tumor microenvironment in uveal melanoma

**DOI:** 10.1038/s41598-022-05599-9

**Published:** 2022-01-31

**Authors:** Feng Zhang, Yan Deng, Dong Wang, Shuai Wang

**Affiliations:** 1 Department of Ophthalmology, Jing Zhou Central Hospital, The Second Clinical Medical College, Yangtze University, Jing Zhou, 434020 Hubei China; 2 Department of Hepatobiliary Surgery, Jing Zhou Central Hospital, The Second Clinical Medical College, Yangtze University, Jing Zhou, 434020 Hubei China; 3 Department of Thoracic Surgery, Shandong Provincial Hospital Affiliated to Shandong First Medical University, Jinan, 250021 Shandong China

**Keywords:** Cancer, Computational biology and bioinformatics, Drug discovery, Genetics

## Abstract

The present study aimed to construct a pyroptosis-related gene signature in uveal melanoma (UM) patients. Patients from The Cancer Genome Atlas (TCGA) served as the training cohort, whereas patients (GSE22138) from Gene Expression Omnibus (GEO) served as the validation cohort. Using the Kaplan–Meier (KM) method, univariate analysis, and least absolute shrinkage and selection operator (LASSO) Cox regression, A five pyroptosis-related gene signature was constructed in the training cohort. Patients were divided into high- and low-risk groups. Survival analysis showed that patients in the high-risk group had a shorter survival time. Risk and survival analysis, time-independent receiver operating characteristic (ROC) curve analysis and principal component analysis (PCA) validated that the prognostic signature had greater predictive value in both cohorts. Multivariate analysis proved that the risk score was an independent prognostic factor. Functional analysis showed that the expressed genes in the high-risk group were most abundant in immunological repose-related and tumor-related signaling pathways. Single-sample gene-set enrichment analysis (ssGSEA) revealed that the different risk groups were associated with the tumor microenvironment. Moreover, the predictive signature could help patients be better matched to immunotherapy and targeted treatments. In conclusion, the pyroptosis-related gene signature associated with the tumor microenvironment maybe a reliable tool for predicting the prognosis of UM patients.

## Introduction

Uveal melanoma (UM) is the second most frequent melanoma subtype, representing 5% of all melanomas, and the most prevalent primary intracellular cancer, accounting for 85–95% of all ocular melanoma cases^1–3^. Although early treatment approaches such as radical surgery and radiotherapy have produced great local tumor control, up to 50% of patients would eventually die of the disease resulting from the high liver metastasis rate^4,5^. Despite substantial improvements in early diagnosis and multimodal therapies such as adoptive T cell therapy and immune checkpoint inhibitors, the survival benefit remains limited^6–8^. Furthermore, a growing number of studies have gradually revealed the prognostic relevance of genes and pathways for UM, but the prognosis remains poor mainly due to the high heterogeneity^9,10^. As a result, credible predictive and prognostic biomarkers must be discovered to improve risk prediction and guide tailored therapy.

Pyroptosis is a unique inflammatory form of programmed cell death characterized by cellular swelling and many bubble-like protrusions produced by caspase-1/4/5/11 activation by certain inflammasomes^11^. Pyroptosis causes cell swelling, plasma membrane lysis, chromatin fragmentation, and the release of intracellular pro-inflammatory components such as inflammatory vesicles, gasdermin proteins, and pro-inflammatory cytokines under adverse circumstance^12^. Pyroptosis was initially discovered to be an effective infection-fighting mechanism. However, the relevance of pyroptosis in tumor development is unclear. On the one hand, as a sort of programmed cell death, pyroptosis has been shown to reduce the onset and progression of tumor^13^. On the other hand, pro-inflammatory contents produced by pyroptosis progressions, such as inflammatory vesicles, gasdermin proteins, and pro-inflammatory cytokines, may accelerate the transformation of normal cells into tumor cells^14^. Moreover, a growing number of studies demonstrated that pyroptosis-related various signaling pathways and inflammatory components were significantly associated with cancer progression and prognosis^15,16^. Novel potential targets and chemotherapeutic therapies involving pyroptosis-related genes have also been studied by increasing research^17,18^. As research has progressed, the role of pyroptosis in malignancies has grown more prominent^19^. Investigating the role of pyroptosis in cancer could hold much promise.

Pyroptosis has a dual role in tumor formation and antitumor action. However, its specific functions in UM have been less uncovered. Using a comprehensive analysis of microarray data from The Cancer Genome Atlas (TCGA) and Gene Expression Omnibus (GEO) databases, the current study aimed to investigate the detailed prognostic value of pyroptosis-related genes, establish a prognostic pyroptosis-related gene signature, and study the correlation of pyroptosis with the tumor immune microenvironment as well as the treatment effectiveness of immunotherapy and targeted therapy.

## Results

### Construction of a pyroptosis-related gene prognostic signature in the TCGA cohort

**Figure 1:**
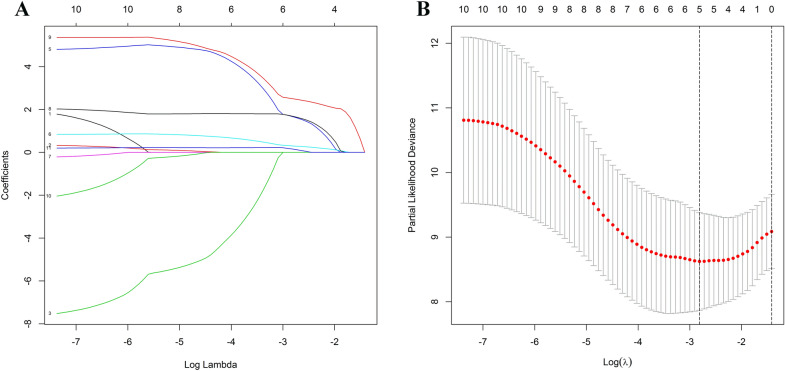
Construction of the risk signature in UM patients in the training cohort. Parameters were going to zero with the penalty (lambda) increasing in the objective function of the LASSO (**A**). 1, *AIM2*; 2, *CASP1*; 3, *CASP5*; 4, *CASP8*; 5, *GSDMC;* 6, *GSDMD*; 7, *IL18*; 8, *IL6*; 9, *NLRP6*; 10, *NOD2*; 11, *PLCG1*. Selection of the optimal variables (lambda) in the LASSO model (**B**). The figure was performed using R software (version 3.3.1, Vienna, Austria, https://www.r-project.org/).

According to Kaplan–Meier survival analysis and univariate Cox proportional hazards model, eleven pyroptosis-related genes may have prognostic significance (Fig. S1 and Table S1). Following that, LASSO regression analysis further identified five appropriate candidates from above eleven prognosis-related genes (Fig. 1). According to the expression level and risk coefficient of these five pyroptosis-related genes, a prognostic signature was constructed as follows: Risk Score = (1.730 × expression level of *GSDMC*) + (0.318 × expression level of *GSDMD*) + (1.735 × expression level of *IL6*) + (2.540 × expression level of *NLRP6*) + (0.186 × expression level of *PLCG1*). The five pyroptosis-related genes listed above were risk genes. Each UM patient was assigned a risk score based on the prognostic signature.

### Internal validation of the prognostic signature in the TCGA cohort

**Figure 2:**
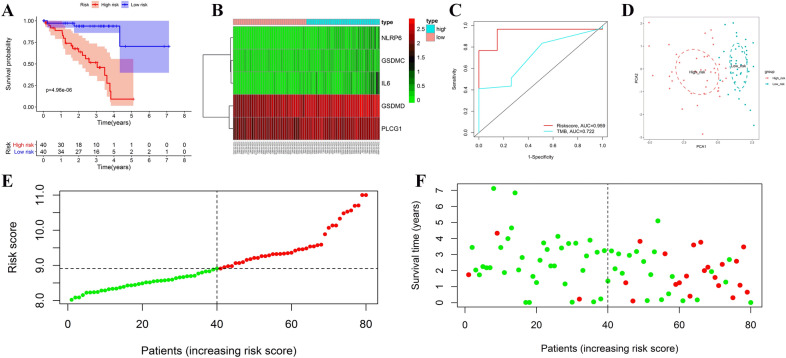
Validation of the prognostic signature in the training group. Kaplan–Meier (KM) curve compares the overall survival between low- and high-risk groups (**A**). Heatmap of differentially expressed pyroptosis-related genes (**B**). Time-dependent ROC curves of the risk score and TMB (**C**). PCA plot based on the risk score; The red dots represent high-risk patients, whereas the blue dots represent low-risk patients (**D**). Distribution of risk scores between low- and high-risk groups; The red dots represent high-risk patients, whereas the green dots represent low-risk patients (**E**). Survival status of UM patients in the low- and high-risk groups; The red dots represent deaths, whereas the green dots represent survivals (**F**). The figure was performed using R software (version 3.3.1, Vienna, Austria, https://www.r-project.org/).

Based on the median value of the risk score, all patients in the TCGA cohort were divided into high- or low-risk groups. According to survival analysis, patients in the low-risk category had a greater survival rate than those in the high-risk group (Fig. 2A). The heat map showed the expression levels of each prognostic pyroptosis-related gene between the high- and low-risk groups. In UM, *IL6*, *GSDMC*, and *NLRP6* expression levels were relatively low, whereas *GSDMD* and *PLCG1* expression levels were relatively high. Patients with low-risk scores had lower amounts of risk variables, as illustrated in Fig. 2B. Time-dependent ROC curve analysis curve demonstrated that the pyroptosis risk score had better predictive accuracy than TMB (Fig. 2C). Furthermore, PCA revealed that patients in different risk categories were separated into two groups (Fig. 2D). The scatter dot plot of patients' risk scores in different groups revealed that the high-risk zone had many deaths and that patients in the low-risk zone had a longer overall survival time than those in the high-risk zone (Fig. 2E,F).

### External validation of the prognostic signature in the GEO cohort

**Figure 3:**
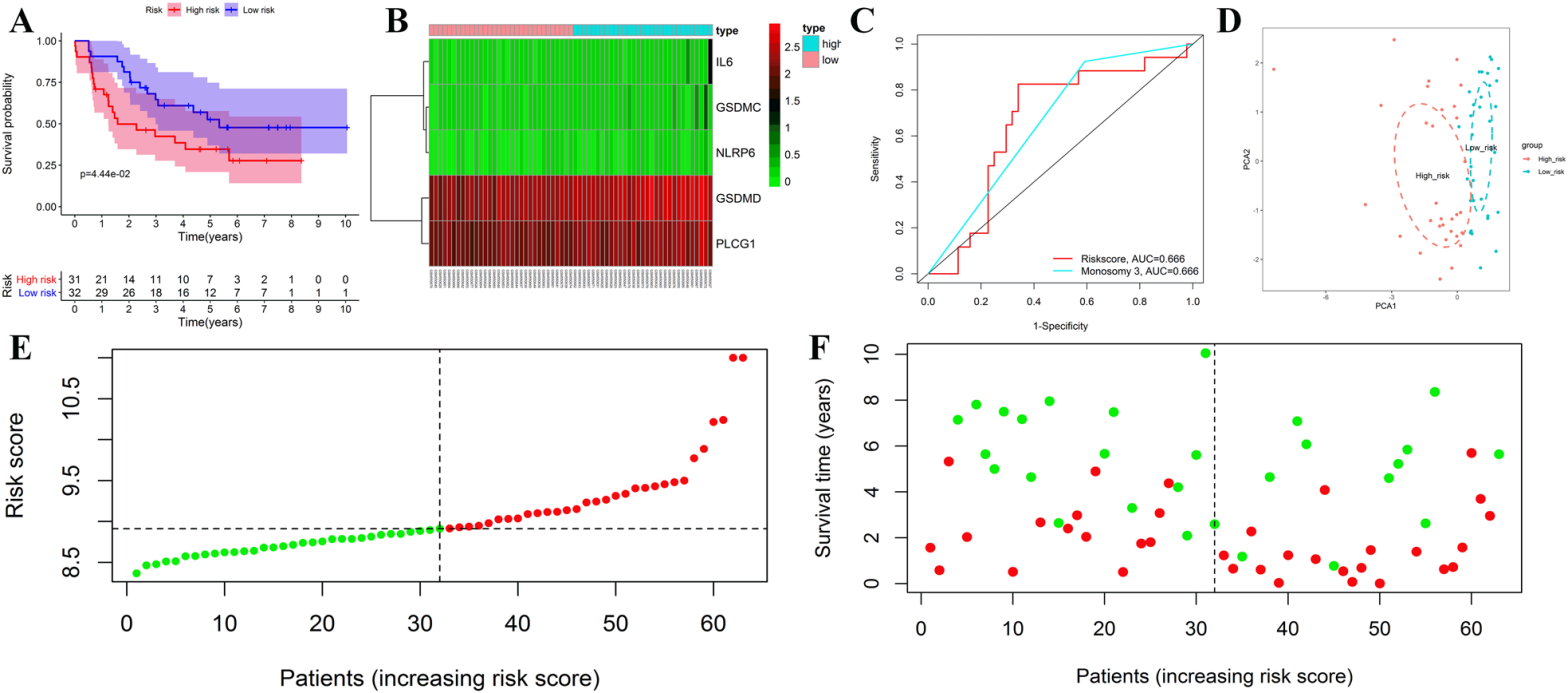
External validation of the risk signature in the validation cohort. KM curve for comparison of metastasis-free survival between low- and high-risk groups (**A**). Heatmap of differentially expressed pyroptosis-related genes (**B**). Time-dependent ROC curves of the risk score and monosomy 3 (**C**). PCA plot based on the risk score; The red dots represent high-risk patients, whereas the blue dots represent low-risk patients (**D**). Distribution of risk scores between low- and high-risk groups; The red dots represent high-risk patients, whereas the green dots represent low-risk patients (**E**). Survival status of UM patients in the low- and high-risk groups; The red dots represent deaths, whereas the green dots represent survivals (**F**). The figure was performed using R software (version 3.3.1, Vienna, Austria, https://www.r-project.org/).

The prognostic signature's prediction potential for metastasis-free survival was next tested in the GEO database's independent validation cohort (GSE22138). The median value of the risk score was used to divide patients into low- or high-risk groups. KM survival curve showed that the low-risk group had less metastasis than the high-risk group, and patients’ metastasis-free survival time in the low-risk group was likewise longer (Fig. 3A). The expression levels of each gene were comparable to those in the TCGA cohort (Fig. 3B). Time-dependent ROC curve analysis validated that the pyroptosis risk score and monosomy 3 had the same predictive accuracy. (Fig. 3C). PCA revealed a good distinction between the high- and low-risk groups, as shown in Fig. 3D. In Fig. 3E, the distribution of patients' risk ratings in different categories was ranked. The scatter dot plot further reveals that patients are more likely to develop metastases as the score increases (Fig. 3F). The distribution of risk scores and the scatter dot plot revealed patients in the low-risk group had fewer deaths and a longer metastasis-free survival time than those in the high-risk group.

### Analysis of the prognostic signature's correlations with clinicopathological characteristics in the TCGA cohort

**Figure 4:**
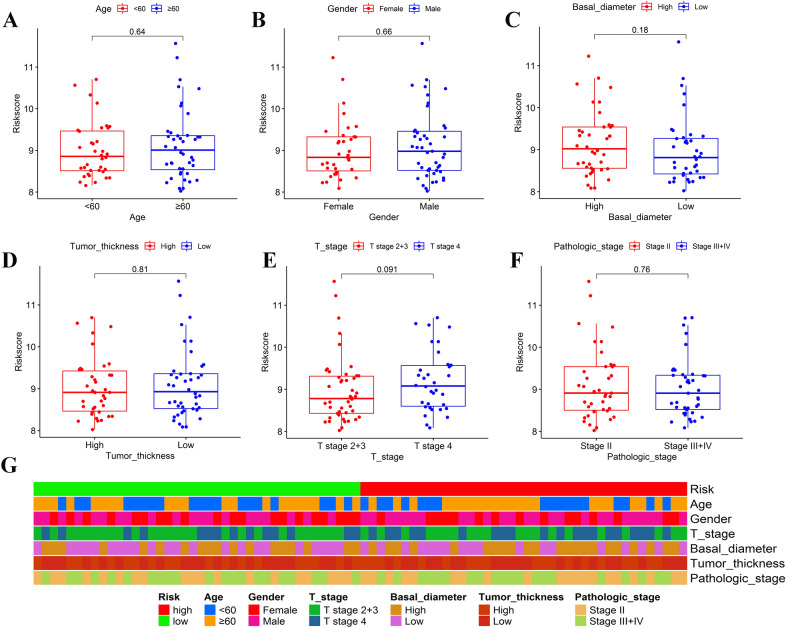
Correlation analysis of the risk score with clinicopathological features. Boxplots of different groups stratified by age (**A**), gender (**B**), basal tumor diameter (**C**), tumor thickness (**D**), tumor T stage (**E**), and pathologic stage (**F**). Heatmap for the connections between clinicopathologic features and the risk groups (**G**). The figure was performed using R software (version 3.3.1, Vienna, Austria, https://www.r-project.org/).

Correlations between the prognostic signature and clinicopathological features were explored in the TCGA cohort. Figure 4 demonstrated that the risk score raised as the T stage progressed, implying that the prognostic signature might be linked to tumor growth. However, the risk score was statistically similar in different subgroups stratified by other clinicopathological factors.

### The pyroptosis-related gene prognostic signature independent of clinicopathological parameters in both cohorts

**Figure 5:**
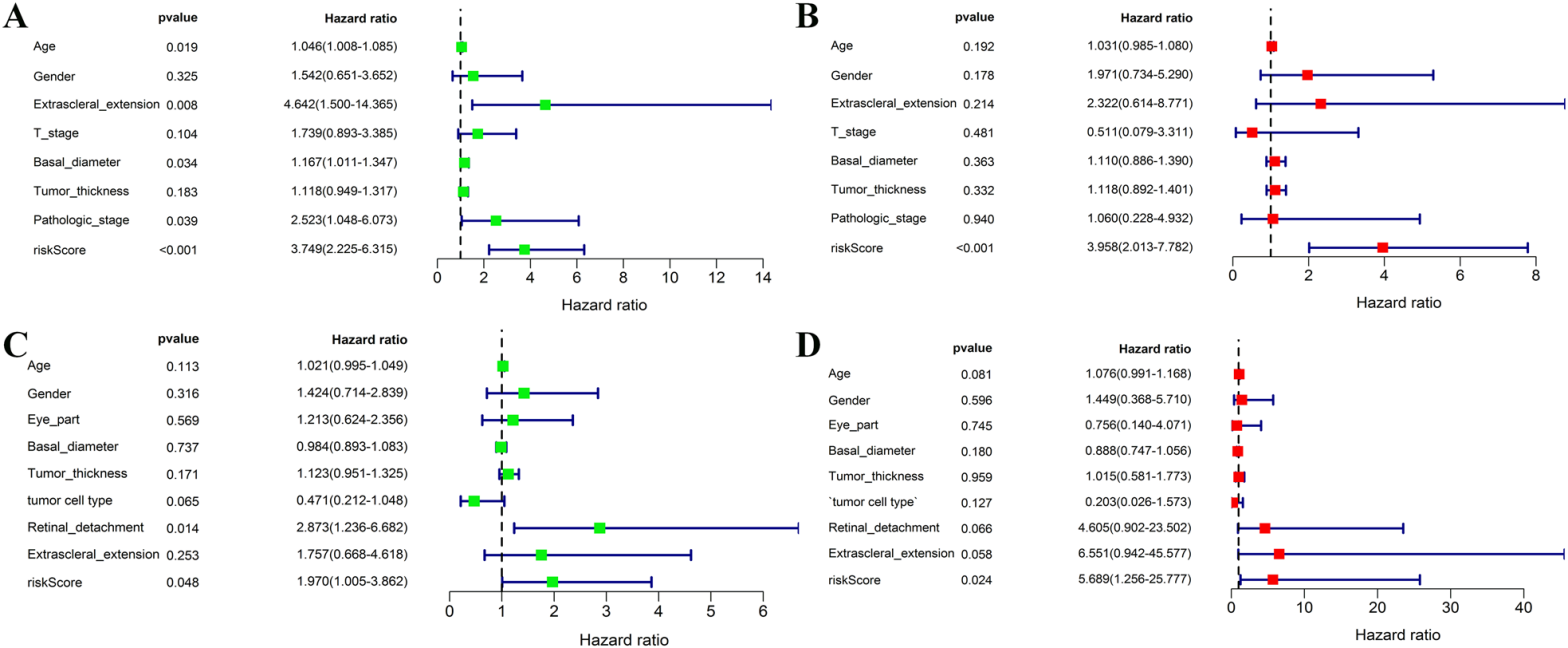
Univariate and multivariate Cox regression analyses for the risk score combined with the clinical characteristics. Univariate analysis for the training cohort (**A**). Multivariate analysis for the training cohort (**B**). Univariate analysis for the validation cohort (**C**). Multivariate analysis for the validation cohort (**D**). The figure was performed using R software (version 3.3.1, Vienna, Austria, https://www.r-project.org/).

We used univariate and multivariate Cox proportional hazards regression analysis in both TCGA and GEO cohorts to see if the prognostic signature's predictive efficacy was independent of clinicopathological characteristics. Univariate analysis revealed the risk score was a prognostic factor [HR 3.749; 95% CI 2.225–6.315; and HR 1.970; 95% CI 1.005–3.862, Fig. 5A,C]. Multivariate analysis further revealed that the risk score was an independent predictor for UM patients in both cohorts after controlling for other confounding factors [HR 3.958; 95% CI 2.013–7.782; and HR 5.689; 95% CI 1.256–25.777, Fig. 5B,D].

### Functional analysis

**Figure 6:**
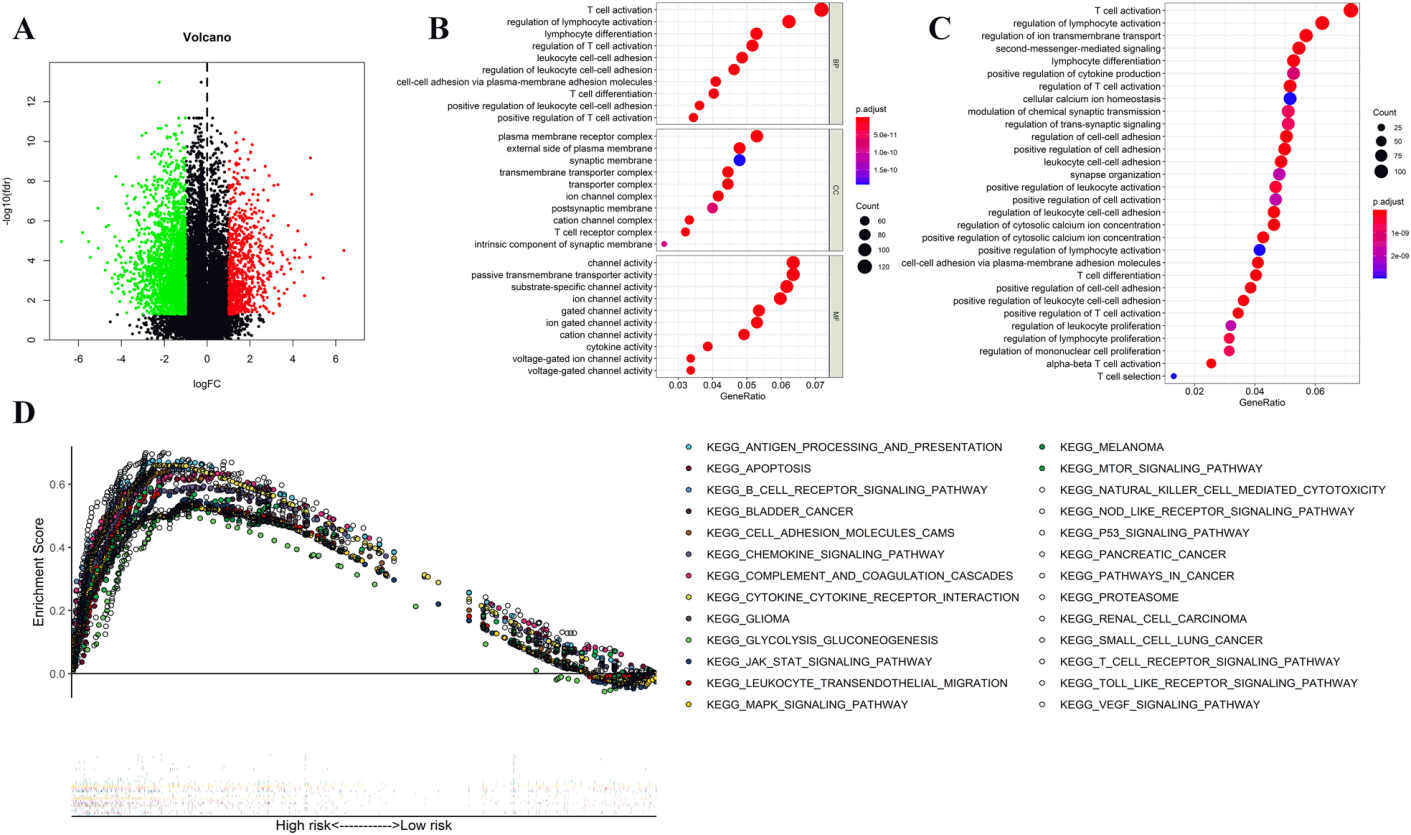
Function analysis. Volcano plot showing the differentially expressed genes between the low- and high-risk groups (**A**). Bubble graph for Gene Ontology (GO) enrichment (**B**). Bubble graph for Kyoto Encyclopedia of Genes and Genomes (KEGG) enrichment (**C**). Gene set enrichment analysis (GSEA) of the differentially expressed genes between high- and low-risk groups; The lower section represents that the further to the left the patient's risk score increases, and the further to the right the patient's risk score decreases (**D**). The figure was performed using R software (version 3.3.1, Vienna, Austria, https://www.r-project.org/).

Differentially expressed genes between the high- and low-risk groups were selected in the TCGA cohort. The volcano plot depicted differentially expressed genes (shown in Fig. 6A). GO and KEGG pathway studies were done. The findings showed those differentially expressed genes were mainly involved in the immunological response, inflammatory cell chemotaxis, chemokines, channel activity, and cytokine activity, as well as passive transmembrane transporter activity (Fig. 6B,C).

Subsequently, GSEA analysis demonstrated that those differentially expressed genes between the were predominantly enriched in immune response-related, chemokine-mediated, and tumor-related signaling pathways. Furthermore, the high-risk group’s changed gene sets were directly linked to malignant tumors (Fig. 6D).

### Comparison of the immune activity and tumor microenvironment between the low- and high-risk subgroups in the TCGA cohort

**Figure 7:**
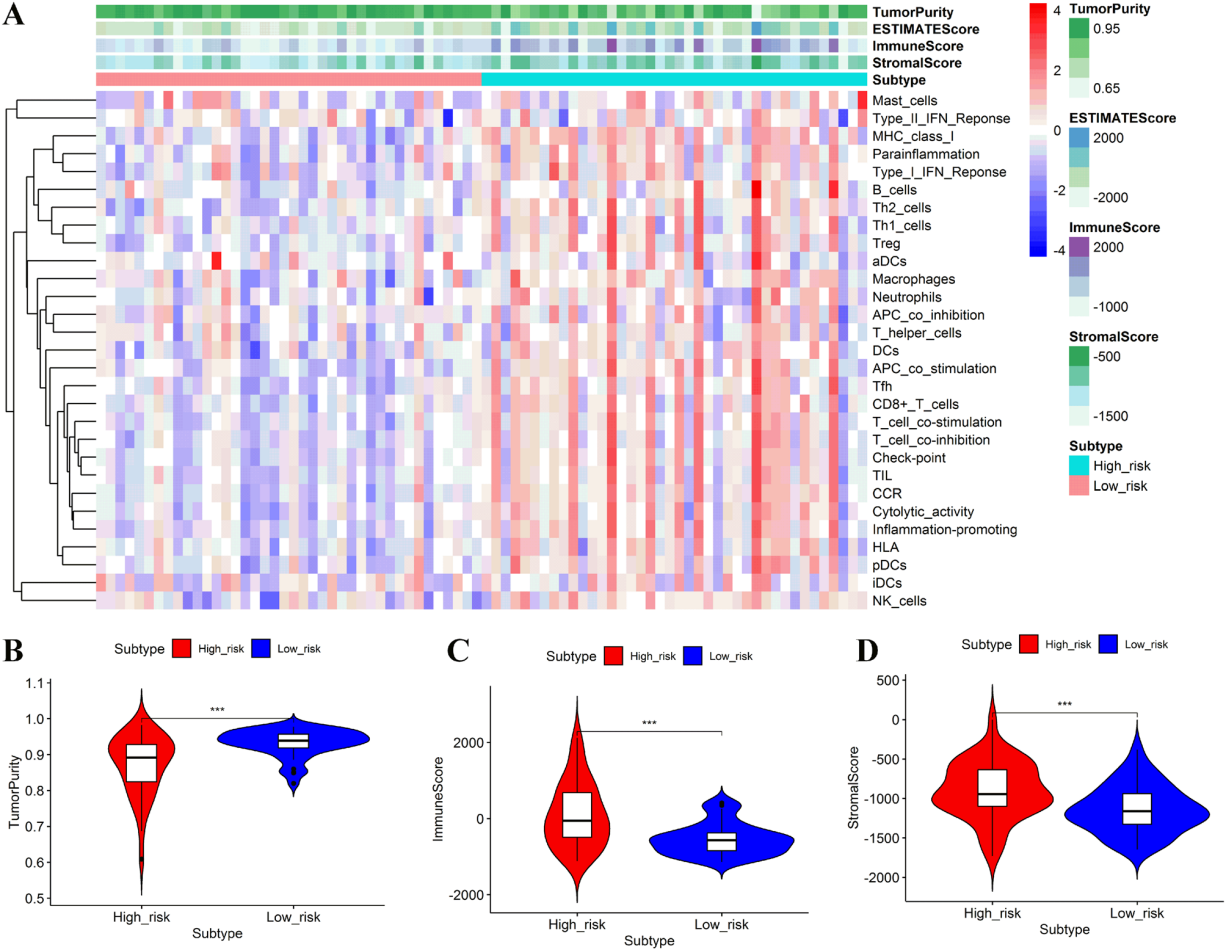
Evaluation of immune cell infiltration level, tumor purity, and stromal content. Hierarchical clustering between high- and low-risk groups (**A**). Comparison of tumor purity between the high- and low-risk groups (**B**). Comparison of the immune cell infiltration scores between the high- and low-risk groups (**C**). Comparison of stromal scores between the high- and low-risk groups (**D**). The figure was performed using R software (version 3.3.1, Vienna, Austria, https://www.r-project.org/).

Subsequently, the immune activity and tumor microenvironment were compared between the low- and high-risk groups in the TCGA cohort. Figure 7 showed that patients in the high-risk group generally had a higher infiltration of immune cells than those in the low-risk group. Moreover, 13 immune pathways showed lower activity in the low-risk group than in the high-risk group. When comparing the tumor purity, immune scores, and stromal scores between the two risk subgroups, we found the immune scores and stromal score were significantly lower in the low-risk group, while the tumor purity obtained opposite trend, with tumor purity decreasing from the low-risk subgroup to the high-risk subgroup (Kruskal–Wallis test, *P* < 0.001).

### Effectiveness prediction of immunotherapy and targeted therapy with the prognostic signature

**Figure 8:**
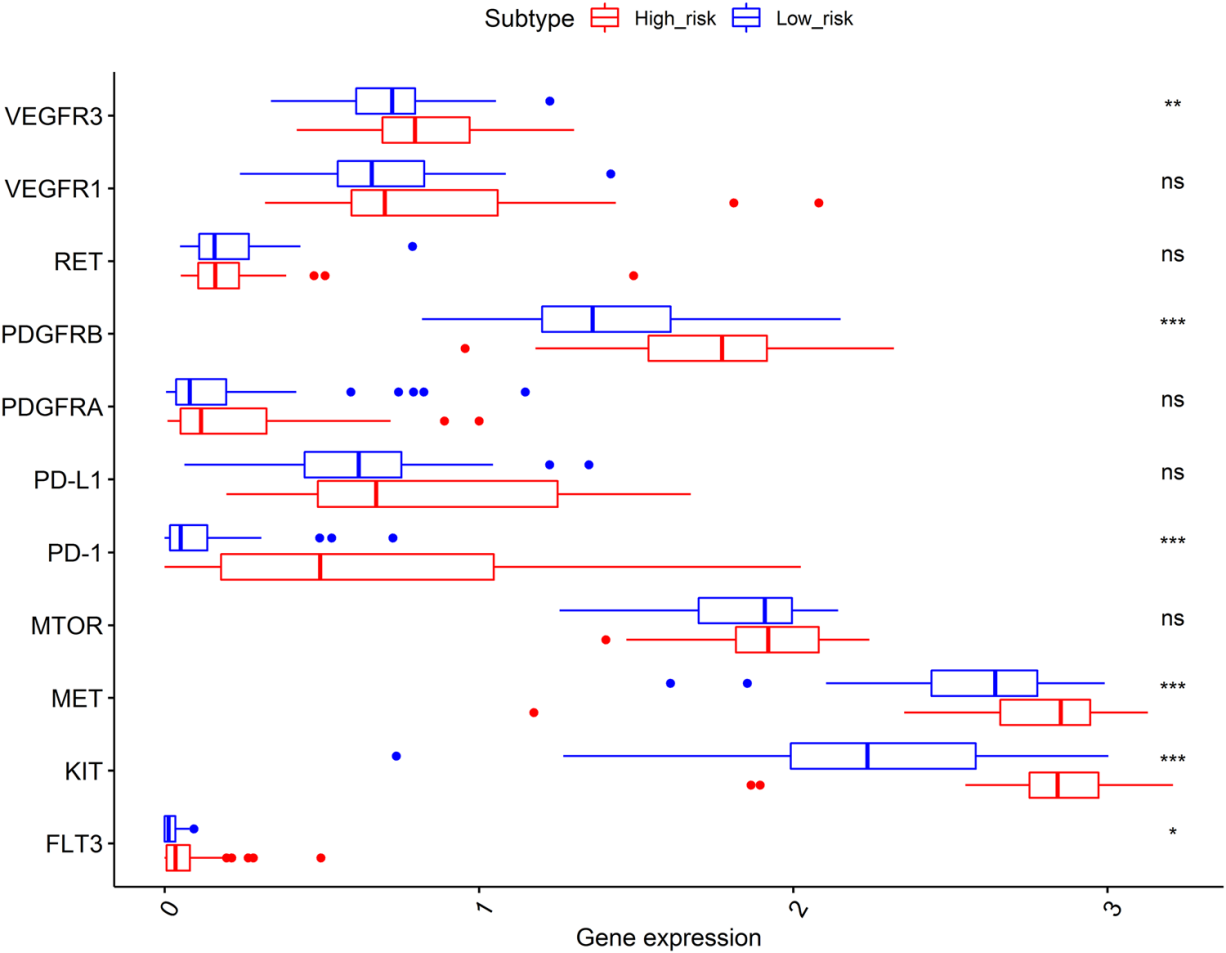
Comparison of the expression levels of immunotherapy and targeted treatment targets between high- and low-risk groups. The figure was performed using R software (version 3.3.1, Vienna, Austria, https://www.r-project.org/).

The immunotherapy and targeted therapy mRNA expression levels were compared between the high- and low-risk groups in Fig. 8. According to the findings, *VEGFR3*, *PDGFRB*, *PD-1*, *MET*, *KIT*, and *FTLT3* mRNA expression levels were considerably more significant in the high-risk group than in the low-risk group. Therefore, immunotherapy and targeted therapy medicines targeting *VEGFR3*, *PDGFRB*, *PD-1*, *MET*, *KIT*, and *FTLT3* may be more effective in UM patients with higher risk scores.

## Discussion

UM is one of the most prevalent primary intracellular malignant tumors in adults, and it is primarily derived from melanocytes in the uveal tract^20^. Undoubtedly, surgical excision of the tumor and surrounding tissue is the primary treatment^21^. In clinical practice, conservative postoperative treatments such as radiation and multimodal therapies are commonly used^22–24^. Despite significant improvements in multimodal therapy, the survival benefit remains limited^25^. Several prognostic variables, such as histological grade, tumor stage, and abnormal genes expression, were well established, the survival advantages were significantly different even among UM patients receiving the same treatment in the same settings^26,27^. Therefore, screening high-risk patients to receive adequate treatment is particularly significant, whereas low-risk patients could receive appropriate treatment to avoid long-term toxicity and morbidity. It is particularly important to construct a prognostic signature based on abnormal gene expression to stratify UM patients at risk to further assist doctors in therapy optimization and clinical decision-making.

Pyroptosis, also known as cellular inflammatory necrosis, is a novel form of programmed cell death. Pyroptosis shares several properties with apoptosis, such as nuclear condensation, DNA damage, and caspase independence^28^. However, pyroptosis has particular morphological features distinguished from other programmed cell deaths. Similar to necrosis, the formation of pores disrupts the balance of ion gradients on both sides of the cell membrane, resulting in water inflow, cell swelling, cell membrane rupture, and the release of pro-inflammatory mediators during pyroptosis^29,30^. Hence, pyroptosis is also referred to as inflammatory “necrosis”^31^. The close relationships between pyroptosis and human diseases, including malignant tumors, have been gradually studied. Tumorigenesis is related to various factors, including the activity of proto- and antioncogenes, the immune microenvironment, oxidative stress, and chronic inflammation. Long-term exposure to tissues and/or cells in the inflammatory environment increases the risk of cancer. Pyroptosis activation causes the release of the inflammatory mediators IL-1 and IL-18, which promote cancer progression in various ways^32^. Hence, pyroptosis might have a dual role in tumor cell growth inhibition or promotion. Researchers have attempted to integrate pyroptosis with various tumor treatments to cure malignancies by regulating pyroptosis and decreasing tumor cell proliferation, migration, and invasion^33^. Exploring the involvement of pyroptosis in the tumor could thus provide a fresh viewpoint on cancer treatment. However, it is unknown if pyroptosis-related genes are associated with prognosis or whether they may be used to assess the success of targeted therapy and immunotherapy in UM.

In the study, we used the expression profile of UM patients’ tumor tissue from the TCGA and GEO databases to investigate the prognostic usefulness of pyroptosis-related genes and develop a prognostic signature. We firstly obtained the expressions of 33 pyroptosis-related genes by analyzing high-throughput RNA transcriptome data. Subsequently, we gradually constructed a five-gene risk signature using the KM method, univariate Cox regression, and LASSO Cox regression in the TCGA cohort. Internal validation confirmed the prognostic signature’s robust predictive potential and perfect accuracy. Furthermore, the predictive potential of the prognostic signature was further tested in an independent cohort from the GEO dataset, confirming the substantial predictive power of the UM prognosis that our signature possessed. When comparing the predictive ability of the risk score with current genetic prognostication methods, we found our risk score had better predictive accuracy (Table S3). Subsequently, we discovered that the risk score could be linked to tumor progression. The explanation supported the findings that improper pyroptosis contributed to a poor tumor microenvironment, allowing the malignant cell to proliferate, invade, and migrate quickly as the tumor advanced. The prognostic signature was proven to be a prognostic factor independent of all clinicopathological parameters in subsequent univariate and multivariate Cox regression analyses. The findings revealed that the prognostic pyroptosis-related gene signature has significant therapeutic potential. Pyroptosis in UM might be a potential area of further investigation.

Functional analyses revealed that differentially expressed genes in the high-risk group were mostly involved in immune response, inflammatory cell chemotaxis, chemokines, channel and cytokine activity, and passive transmembrane transporter activity. GSEA analysis showed that the differentially expressed genes between the high- and low-risk groups were mainly enriched in immune response-related, chemokine-mediated, and tumor-related signaling pathways. Moreover, the high-risk group's changed gene sets were directly linked to malignant tumors. These findings concluded that pyroptosis might influence carcinogenesis, development, and metastasis by changing the composition of the tumor microenvironment. Subsequently, we compared immune cell infiltration and antitumor immune activities between the high- and low-risk subgroups. The results showed that patients in the low-risk group had less robust immune cell infiltration and antitumor immune activities than those in the high-risk group, indicating that the high-risk group's immune functions were impaired overall. Low-risk patients had better clinical results than high-risk patients, which could be explained by strong antitumor immune activation. An increasing number of studies confirmed that the density of tumor-infiltrating lymphocytes is positively associated with survival prognosis in various types of malignancies. The current study discovered that the immune and stromal scores were significantly lower in the low-risk group, whereas tumor purity had the opposite pattern. The findings also suggested that an unfavorable tumor microenvironment could be blamed for high-risk UMs' poor prognosis and low levels of antitumor immunity.

Immunotherapy and targeted therapy are currently under investigated. Recent studies have attempted to integrate pyroptosis with other tumor treatments, such as immunotherapy and targeted therapy, as well as to cure tumor by regulating pyroptosis. As a result, the pyroptosis-related signature classification may aid in the stratification of UM patients in order to identify those who respond to immunotherapy and targeted therapy. Our finding found that *VEGFR3*, *PDGFRB*, *PD-1*, *MET*, *KIT*, and *FTLT3* mRNA expression levels were considerably greater in the high-risk group than in the low-risk group. As a result, the findings suggested that UM patients with higher risk scores may respond better to immunotherapy and targeted therapy medicines targeting *VEGFR3*, *PDGFRB*, *PD-1*, *MET*, *KIT*, and *FTLT3.* Hence, exploring the effect of pyroptosis on UM might provide a new theoretical basis for the further treatment of UM.

Five pyroptosis-related genes were included in the prognostic signature: *GSDMD*, *GSDMC*, *IL6*,* NLRP6*, and *PLCG1*. To our knowledge, this was the first comprehensive discovery of pyroptosis-related genes to develop a prognostic signature to predict clinical outcomes in UM patients. The activities of these genes in some types of malignancies, including UM, have been investigated in detail. *GSDMD*, a central member of the gasdermin family, is generally in a state of autoinhibition. *GSDMD* releases the N-terminal fragment (GSDMD-cNT) after caspase cleavage, causing the cells to expand until they rupture, showing that *GSDMD* is the pyroptosis executor^34^. Increasing studies suggested that elevated *GSDMD* expression was important for cancer invasion, metastasis, and prognosis^17,35^. Our study verified that UM patients with high *GSDMD* expression had poor overall survival time. *GSDMC*, another member of the gasdermin family, is mapped at 8q24.1-8q24.2 and was initially detected at both differentiating and differentiated epithelium cells in the upper gastrointestinal tract^31^. Miguchi et al.^36^ demonstrated that *GSDMC* was upregulated in colorectal cancer, and its overexpression enhanced cell proliferation and xenograft tumor growth. *IL6*, a main inflammatory cytokine, participates in the poor tumor microenvironment, promoting tumorigenesis and cancer development. Similar to previous studies, high expression of *IL-6* was significantly associated with poor survival outcomes in UM^37^. Nod-like receptor pyrin domain-containing protein 6 (*NLRP6*), belonging to the nod-like receptor (*NLR*) family of pattern recognition receptors, consists of an N-terminal pyrin domain (PYD), a central NOD domain, and C-terminal leucine-rich repeats (LRRs)^38^. *NLRP6* is known as a component of the inflammasome and participates in inflammasome signaling^39^. Downregulated *NLRP6* expression in gastric cancer was significantly associated with overall survival^40^. *NLRP6* appeared to be a cancer-promoting gene in our investigation, as it contributed to shorter patient survival. Given the limited data from UM and the often-conflicting results in different tumors, our results regarding *NLRP6* provided some insights for further studies, especially in UM. Phospholipase C gamma 1 (*PLCG1*) affects cell growth, differentiation, and apoptosis by mediating the receptor tyrosine kinase (RTK)-mediated signal transduction pathway. A prior study showed that *PLCG1* activation was an essential requirement for the continued growth of small cell lung cancer^41^. We found that high *PLCG1* expression was significantly related to poor survival outcomes among UM patients. However, the roles of these genes, except *IL-6*, have rarely been investigated in UM. Understanding pyroptosis and related genes’ function in carcinogenesis and tumor progression might be a potential area of further investigation.

We looked at the predictive value of pyroptosis-related genes as a preliminary study, which could provide a theoretical foundation for further research. However, our study has several flaws. First, our risk signature was only pyroptosis-related genes and did not represent other potential gene transcription expression profiles correlated to OS in UM. In addition, we were unable to explore the exact mechanism of pyroptosis-related genes. The limitation deserves further in-depth studies.

## Conclusion

In conclusion, the pyroptosis-related gene prognostic signature developed was a reliable tool for predicting the prognosis of UM patients. The prognostic characteristic could aid our understanding of pyroptosis' function in carcinogenesis and tumor progression. The prognostic signature could be used to stratify patients at risk based on their prognosis, assisting doctors in therapy optimization and clinical decision-making. Furthermore, exploring the effect of pyroptosis on UM might be a potential area of further investigation.

## Materials and methods

### Datasets

Microarray datasets, including the raw RNA-sequencing and related clinical data information including age, gender, basal tumor diameter, tumor thickness, T stage, pathologic stage, survival status, and survival time of UM patients, were freely downloaded from The Cancer Genome Atlas (TCGA) Database (https://portal.gdc.cancer.gov/) on March 31, 2021. The cohort included 414 cancer tissue samples. GSE22138, an independent cohort of UM patients, was chosen for external validation from the Gene Expression Omnibus (GEO) (https://www.ncbi.nlm.nih.gov/geo/). The cohort included 63 cancer tissue samples. All methods were performed in accordance with the relevant guidelines and regulations.

Tumor mutational burden (TMB), the mutation density of tumor genes, is the number of mutations per million bases in tumor tissue, including base substitutions, gene insertion, and gene coding and deletion errors. It is also defined as the average number of mutations in the tumor genome. Mutation frequency for each tissue could be calculated using the Masked Somatic Mutation data (Varscan. Somatic. Maf) from the TCGA database. The 38 Mb is routinely taken based on the length of the human exon, so TMB is equal to the total mutation frequency/38. Hence, TMB was calculated by dividing the total number of mutations by the size of the coding region of the target. TMB evaluation was conducted by the ‘mafools’ R package.

Because all of the data were obtained directly from the public source, no protocol from the ethics committee was required.

### Pyroptosis-related genes collection

Thirty-three pyroptosis-related genes were extracted from prior studies in Table S2^19–23^. The expression levels of 33 pyroptosis-related genes were retrieved from the TCGA and GEO datasets. The procedure was performed by the Perl programming language (Version 5.30.2, http://www.perl.org).

### Construction of the pyroptosis-related gene prognostic signature

First, the Kaplan–Meier (KM) method and univariate Cox regression analysis were performed by the “survival” R package to identify prognosis-related and pyroptosis-related genes with both significant *P* values less than 0.050 in the TCGA cohort. Second, to avoid overfitting, LASSO regression analysis was performed by the “glmnet” R package to identify appropriate candidates from prognosis-related genes. Subsequently, appropriate candidate genes and their coefficients were retained based on the ideal penalty parameter (λ) value. Finally, a prognostic pyroptosis-related gene signature was constructed by the sum of the value of coefficients multiplied by the genes’ expression.

### Validation of the prognostic pyroptosis-related gene signature

According to the prognostic signature, each UM patient in the TCGA and GEO groups received a risk score. Based on the median value of the risk score, UM patients were divided into high- or low-risk groups. A lower risk score denoted a low-risk situation, whereas a larger risk score denoted a high-risk situation. The prognostic signature's prediction efficacy was then tested in both cohorts.

K-M curve was drawn using the “survival” R package to compare the survival difference of the high- and low-risk groups, and a two-sided log-rank test assessed the difference between the two groups. The "pheatmap" R package drew heat map to show the different expression levels of prognostic pyroptosis-related genes in high- and low-risk groups. The specificity and sensitivity of the prognostic signature were assessed using a 3-year time-dependent receiver operating characteristic (ROC) curve analysis using the “survival”, “survminer”, and “timeROC” R packages, and the area under the ROC curve (AUC) was used to evaluate the signature's prognostic accuracy. The AUC ranges from 0.5 (no predictive power) to 1 (perfect prediction). The unique ability was investigated using principal component analysis (PCA) based on the five pyroptosis-related gene signatures. The “ggplot2” R packages were employed to perform the PCA analysis. Finally, the detailed correlations of death states with risk scores were visualized using the distribution of patients' risk scores and scatter dot plots.

### Independent prognostic analysis of the risk score

We merged the clinicopathological information with the patients’ risk scores. Correlations of the risk score with clinicopathological characteristics were further investigated. Univariate and multivariate Cox proportional hazards regression analyses were performed by the “survival” R package to see if the prognostic signature's predictive efficacy was independent of clinicopathological characteristics.

### Functional analysis

The “limma” R package was used to identify differentially expressed genes between the high- and low-risk groups based on the following criteria: |logFC| > 1 and an adjusted *P*-value < 0.050. Based on these differentially expressed genes, Gene Ontology (GO), and Kyoto Gene and Genomic Encyclopedia (KEGG) analyses were conducted by applying the "org.Hs.eg.db", "colorspace", "stringi", "ggplot2", "dose", "clusterProfiler", and "enrichplot" R packages. Subsequently, underlying mechanisms were investigated within the “Molecular Signatures Database” of c2.cp.kegg. v6.2. Symbols through gene set enrichment analysis (GSEA) (software 4.1) with a Java program. The random sample permutation number was set as 1,000, and the significance threshold was *P*-value < 0.050.

### Evaluation of immune cell infiltration level, tumor purity, and stromal content

We analyzed 29 immune-associated gene sets representing diverse immune cell types, functions, and pathways in the TCGA cohort. The immune cell infiltration level (immune score), tumor purity (tumor score), and stromal content (stromal score) for each UM sample were investigated using ESTIMATE^42^. The single-sample gene-set enrichment analysis (ssGSEA) score was used to quantify the activity and enrichment level of immune cell types, functions, and pathways applying the "limma", "GSVA", and "GSEABase" R packages in all samples. Heatmap result was exhibited by the "pheatmap" R package. The Spearman correlation of the risk score with the immune score, tumor purity, and stromal score was used to evaluate the correlation of pathway activities with immune cell infiltration levels in UM. Wilcoxon rank-sum test was performed to assess the difference between high- and low-risk groups, and the result was exhibited by the "ggpubr" R package.

### Exploration of the associations of the risk score with targets of targeted therapy and immunotherapy

Targeted therapy and immunotherapy have become practical approaches for the treatment of malignant tumors. Pearson's correlation analysis was used to evaluate the associations of the risk score with therapy-related goals. We sought to predict treatment success using our risk score. The therapy targets are listed as follows: programmed cell death 1 (PD-1, also known as *PDCD1*), vascular endothelial growth factor receptor (*VEGFR1*, also known as *FLT1*), Fms-like tyrosine kinase 3 (*FLT3*), vascular endothelial growth factor receptor 3 (*VEGFR3*, also known as *FLT4*), platelet-derived growth factor receptor alpha (*PDGFRA*), platelet-derived growth factor receptor beta (*PDGFRB*), KIT proto-oncogene (*KIT*), ret proto-oncogene (*RET*), MET proto-oncogene (*MET*), programmed cell death ligand 1 (*PD-L1*, also known as *CD274*), and mammalian target of rapamycin (*mTOR*). Those associations were drawn by the "ggpubr" R package, and the difference was evaluated by Wilcoxon rank-sum test.

### Statistical analysis

All statistical analyses and generation of figures were performed by the Perl programming language (Version 5.30.2, http://www.perl.org) or R software 4.0.2 (R Foundation for Statistical Computing, version 4.0.2, Vienna, Austria, http://www.r-project.org/). A two-sided *P-*value < 0.050 was considered to be statistically significant.

## Supplementary Information

Supplementary Information.

## Data Availability

Publicly available datasets from the Cancer Genome Atlas (https://portal.gdc.cancer.Gov/) and International Cancer Genome Consortium (https://icgc.org/) were analyzed in this study. All data supporting the findings of the study are available from the corresponding author on reasonable request. All data in our study are available upon request.

## Abbreviations

UM: Uveal melanoma
TCGA: The cancer genome atlas
GEO: Gene expression omnibus
KM: Kaplan–Meier
LASSO: Least absolute shrinkage and selection operator
ROC: Receiver operating characteristic
PCA: Principal component analysis
ssGSEA: Single-sample gene-set enrichment analysis
AUC: Area under the ROC curve
TMB: Tumor mutational burden
FC: Fold change
*PDCD1*: Programmed cell death 1
*VEGFR1*: Vascular endothelial growth factor receptor
*FLT3*: Fms-like tyrosine kinase 3
*VEGFR3*: Vascular endothelial growth factor receptor 3
*PDGFRA*: Platelet-derived growth factor receptor alpha
*PDGFRB*: Platelet-derived growth factor receptor beta
*KIT*: KIT proto oncogene
*RET*: Ret proto oncogene
*mTOR*: Mammalian target of rapamycin
