# Supplementary material for: Construction and validation of a pyroptosis-related gene signature associated with the tumor microenvironment in uveal melanoma
Source: Sci Rep. 2022 Jan 31;12:1640. doi: 10.1038/s41598-022-05599-9 (PMC8803850; doi:10.1038/s41598-022-05599-9)
Supplement: Supplementary file 1 — Supplementary Information. [file 41598_2022_5599_MOESM1_ESM.docx]

**Supplementary Table S1:** Univariate Cox regression analysis of the pyroptosis-related potential prognostic genes in the TCGA cohort

| Gene | HR | HR.95L | HR.95H | *P* value |
| --- | --- | --- | --- | --- |
| AIM2 | 2.436 | 1.295 | 4.582 | 0.006 |
| CASP1 | 1.783 | 1.2435 | 2.557 | 0.002 |
| CASP5 | 38.316 | 1.827 | 803.657 | 0.019 |
| CASP8 | 3.175 | 1.257 | 8.0184 | 0.014 |
| GSDMC | 3554.980 | 20.310 | 622255.600 | 0.002 |
| GSDMD | 2.835 | 1.585 | 5.069 | <0.001 |
| IL18 | 2.110 | 1.160 | 3.838 | 0.014 |
| IL6 | 19.108 | 4.239 | 86.123 | <0.001 |
| NLRP6 | 107.858 | 11.777 | 987.836 | <0.001 |
| NOD2 | 2.848 | 1.342 | 6.045 | 0.006 |
| PLCG1 | 2.405 | 1.1272 | 5.1325 | 0.023 |

**Supplementary Table S2:** pyroptosis-related genes

| Gene |
| --- |
| AIM2 |
| CASP1 |
| CASP3 |
| CASP4 |
| CASP5 |
| CASP6 |
| CASP8 |
| CASP9 |
| ELANE |
| GPX4 |
| GSDMA |
| GSDMB |
| GSDMC |
| GSDMD |
| GSDME |
| IL18 |
| IL1B |
| IL6 |
| NLRC4 |
| NLRP1 |
| NLRP2 |
| NLRP3 |
| NLRP6 |
| NLRP7 |
| NOD1 |
| NOD2 |
| PJVK |
| PLCG1 |
| PRKACA |
| PYCARD |
| SCAF11 |
| TIRAP |
| TNF |

**Supplementary Table S3:** Compared the predictive ability of the pyroptosis-related riskscore with current genetic prognostication methods.

| Signature | AUC |
| --- | --- |
| Pyroptosis-related gene signature | 0.959 |
| Autophagy-related gene signature^1^ | 0.953 |
| Immune-related gene signature^2^ | 0.911 |
| Ferroptosis-related gene Signature^3^ | 0.874 |

AUC: area under the receiver operating characteristic curve





**Supplementary Figure S1:** Kaplan Meier survival curves for pyroptosis-related genes with prognostic significance in the training group. The figure was performed using R software

**References**

1 Zheng, Z., Zhang, L., Tu, Z., Deng, Y. & Yin, X. An autophagy-related prognostic signature associated with immune microenvironment features of uveal melanoma. Biosci Rep 41, doi:10.1042/BSR20203812 (2021).

2 Gu, C., Gu, X., Wang, Y., Yao, Z. & Zhou, C. Construction and Validation of a Novel Immunosignature for Overall Survival in Uveal Melanoma. Front Cell Dev Biol 9, 710558, doi:10.3389/fcell.2021.710558 (2021).

3 Luo, H. & Ma, C. A Novel Ferroptosis-Associated Gene Signature to Predict Prognosis in Patients with Uveal Melanoma. Diagnostics (Basel) 11, doi:10.3390/diagnostics11020219 (2021).
